# Pharmaceutical Chemistry

**Published:** 1845-04

**Authors:** 


I. The Pharmacopœia of the Royal College of Physicians of Edinburgh. 12mo. pp. 217. Edinburgh, 1839.

The Second Edition of the above, 1841.

II. A Dispensatory, or Commentary on the Pharmacopœias of Great Britain, &c. &c. By *Robert Christison*, M.D. F.R.S.E. Professor of Materia Medica in the University of Edinburgh, Vice-President of the College of Physicians, &c. 8vo. pp. 978. Edinburgh, 1842.

III. The London Dispensatory, &c. &c. By *Anthony Todd Thomson*, M.D. F.L.S. Professor of Materia Medica, Therapeutics and Medical Jurisprudence in University College, London, Fellow of the Royal College of Physicians in London, &c. &c. 8vo. pp. 1317. London, 1844.

[Continued from page 219.]

Without further preface we continue our examination of the Edinburgh Pharmacopœia, and of Dr. Christison's and Dr. Thomson's Dispensatories.

*Ferri Sulphuretum.*—The following are the directions of the Edinburgh College for preparing this compound:—

"Ferri Sulphuretum.
The best Sulphuret of Iron is made by heating an iron rod to a full-white heat in a forge, and rubbing it with a roll of sulphur over a deep vessel filled with water to receive the fused globules of sulphuret which form. An inferior sort, good enough however for pharmaceutic purposes, is obtained by heating one part of sublimed sulphur and three of iron-filings in a crucible in a common fire till the mixture begins to glow, and then removing the crucible and covering it, until the action, which at first increases considerably, shall come to an end."

The process of the Dublin Pharmacopœia, is as follows:—

"Let an iron rod be heated to whiteness in a very strong fire, urged by bellows, and being immediately removed from the fire, let it be applied to a solid mass of sulphur. Let the Sulphuret of Iron received in water be separated from the Sulphur, then dried and kept in close vessels."

These processes are mixed and metamorphosed by Dr. Christison in the following manner:—

"FERRI SULPHURETUM, *E. D.* *Protosulphuret of Iron.* *Sulphuret of Iron.*

PROCESS, *Edin.* Take of

Iron-filings, three parts;

Sublimed sulphur, one part.

Mix them thoroughly; heat the mixture in a covered crucible till it become red hot; remove the crucible from the fire and allow the action to go on without heat.

PROCESS, *Edin. Dub.* A purer sulphuret may be obtained by heating an iron-rod to a white heat in a blacksmith's forge, applying a stick of sulphur to the end of the rod, and collecting in water the fused globules which fall down. These should be freed of sulphur and kept in a close vessel."

Dr. Christison observes, that the Sulphuret which is prepared from iron-filings by one of the Edinburgh processes, contains an excess of iron; this excess is indeed so obviously great that it is singular it should have been directed to be employed. Protosulphuret of iron consists of one equiv. of sulphur 16 + one equiv. of iron 28, and supposing no sulphur to be sublimed or burnt in the operation, the result of it must consist of 44 parts of sulphuret and 20 of iron; so that when 16 of sulphur are heated with 48 of iron, the mass must consist of 44 parts of sulphuret mixed with 20 parts of iron; and when 64 grains are acted upon by dilute sulphuric acid, the gas obtained is a mixture of about 46 cubic inches of sulphuretted hydrogen, and 33 cubic inches of mere hydrogen. It is indeed true that the hydrogen does no absolute mischief, but the escape of this gas, especially when mixed with sulphuretted hydrogen, is always disagreeable; added to which, about four-tenths of the sulphuric acid employed in liberating this mixture of gases is wasted.

Dr. Thomson has entirely omitted the Dublin process; with respect to the Edinburgh formulas, he says, "the result of the first contains an excess of iron: it is only by the first process, carefully executed, that a pure sulphuret is obtained." We need hardly observe that the remark should have been, that the result of the first process contains *no* excess of iron; it is the second which does so.

*Ferrugo* is the preparation which we shall next notice. The directions for obtaining this substance, which we find in the index is translated Rust of Iron, is thus given in the Edinburgh Pharmacopœia.

"FERRUGO.
Take of Sulphate of Iron, four ounces:
Sulphuric Acid (commercial) three fluidrachms and a-half;
Nitric Acid (D. 1380), nine fluidrachms;
Stronger Aqua Ammoniæ three fluidounces and a-half.
Water, two pints;
Dissolve the Sulphate in the water, add the Sulphuric acid, and boil the solution; add then the Nitric acid in small portions, boiling the liquid for a minute or two after each addition, until it acquires a yellowish-brown colour and yields a precipitate of the same colour with ammonia. Filter; allow the liquid to cool; and add in a full stream the Aqua Ammoniæ, stirring the mixture briskly. Collect the precipitate on a calico-filter; wash it with water till the washings cease to precipitate with nitrate of baryta; squeeze out the water as much as possible; and dry the precipitate at a temperature not exceeding 180°.

"When this preparation is kept as an antidote for poisoning with arsenic, it is preferable to preserve it in the moist state, after being simply squeezed."

Dr. Christison, in his Dispensatory, has accidentally substituted three *fluidounces* and a-half of sulphuric acid for three *fluidrachms* and a-half. It will be remembered that, in preparing the Ferri oxidum nigrum, the protoxide of half of the sulphate of iron is converted into sesquioxide by means of nitric acid; and for this purpose, on that occasion, the College direct pure nitric acid of sp. gr. 1·5 to be employed, while in Ferrugo, they order commercial nitric acid of sp. gr. 1·380 to be used for a similar purpose; for this diversity of practice we can discover no reason; the commercial acid is the more economical, and why it should not have been used in both preparations, we are at a loss to conjecture.

We attempted to prepare this medicine in the mode ordered, adding the nitric acid a fluidrachm at a time to the solution of sulphate of iron, and heating it after each addition; we found, however, on employing the test recommended by the College, of the colour of the precipitate yielded by ammonia, that the whole of the protoxide of iron was not converted into sesquioxide until the boiling had been so long continued as that scarcely 16 fluidounces of Solution remained, consequently 24 fluidounces of water were evaporated; as the solution was evidently much too concentrated to be conveniently decomposed, water was added to it to make it up to two pints, or the original measure; on mixing ammonia with a small portion of this diluted solution in a test tube, so gelatinous a precipitate of sesquioxide of iron was formed, that on inverting the tube scarcely a drop of liquid left it.

The quantity of the solution was then, by the addition of water, increased to four pints, and even then the precipitate given by the ammonia was so bulky, that scarcely two pints of clear liquor could be poured off from it, after allowing eighteen hours for the subsidence of the oxide of iron.

It is we think, quite evident, that if any one had attempted to prepare Ferrugo according to the directions of the Pharmacopœia they never would have found a place in it; as to the employment of the stronger instead of the weaker solution of ammonia, we refer to what we have said under the head of Ferri oxidum nigrum.

There are some inconsistencies in the College directions for preparing the three precipitated oxides of iron, which we shall point out. Under Ferri oxidum nigrum the ammonia is to be "immediately" added in a full stream to the mixed and hot solutions of protoxide and peroxide of iron, whereas in Ferrugo the solution of iron, with evident propriety, is directed to be allowed to cool, before admixture with the ammonia.

With respect to the Ferri Oxidum nigrum, the precipitate is directed to be washed, "till the water is scarcely precipitated by solution of nitrate of baryta;" and the precipitated Ferri oxidum rubrum is to be washed "till the water is but litle affected with solution of nitrate of baryta;" whereas,

Ferrugo is to be washed "till the washings cease to precipitate with nitrate of baryta." We need hardly say that, in our opinion, the directions in the last case are unreasonable, for we found that less than 10 drachms of Ferrugo required about four gallons of distilled water to effect the purpose directed.

Dr. Thomson, like Dr. Christison, has given *fluidounces* of sulphuric acid in the formula for Ferrugo instead of *fluidrachms*, and we should suppose that the former had copied the error from the latter, had we not observed the proneness of Dr. Thomson to commit errors of this sort, as shown by his substituting *fluidounces* for *fluidrachms* of nitric acid in the formula for Ferri oxidum nigrum. The directing of fluidounces instead of fluidrachms of sulphuric acid in Ferrugo is not, however, the only grave error which Dr. Thomson has committed in this formula, for he has entirely omitted the nitric acid, so essential to the process; so that we have eight times the proper quantity of one acid directed to be used, and, as if by way of compensation, the whole quantity of another is omitted.

Alluding to this preparation, Dr. Thomson says, "the salt thus formed has a yellowish-brown colour," meaning, we presume, the *oxide* thus formed; and he further states, "its composition, most probably, when properly prepared, is one part of sesquioxide of iron $= 80 + 2$ parts of water $= 18$, making the equiv. 98 ($Fe^2. O^5. + 2 H. O.$)."

Now, how "one part" can be considered as $= 80$, "or 2 parts" as $= 18$, we are at a loss to conjecture, and therefore propose to read one equivalent for one part, and two equivalents for two parts; in the formula, $O^5$ is misstated for $O^3$.

*Ferri Sulphas.*—The Edinburgh formula for this salt is entirely left out of Dr. Christison's Dispensatory; it is as follows in the Pharmacopœia:

"FERRI SULPHAS.
If the Sulphate of iron of commerce be not in transparent green crystals, without efflorescence, dissolve it in its own weight of boiling water acidulated with a little sulphuric acid; filter; and set the solution aside to crystallize. Preserve the crystals in well-closed bottles."

Supposing commercial sulphate of iron to contain, as it usually does, some sesquipersulphate, it would dissolve in water, and any subsalt of the peroxide would be dissolved by the sulphuric acid; this process seems therefore calculated to reproduce the impurity which it is intended to remove; whereas, by adding a little iron, the sesquioxide would be reduced to protoxide, and any copper which the commercial salt might contain would be precipitated by it.

Among the "formulas for ascertaining the requisite purity of the more important articles and their freedom from known adulterations," we find, with respect to sulphate of iron, the following in Dr. Christison's Dispensatory: "TESTS. *Edin.* Entirely soluble: its solution precipitated by excess of ammonia, yields on filtration a fluid which is colourless or very pale blue."

The reader will perhaps scarcely believe that not one word of this statement of the tests is to be found in the Edinburgh Pharmacopœia, yet such is the case, for the tests which it gives are merely the following. "Pale bluish-green crystals, with little or no efflorescence." The question then naturally arises, what could induce Dr. Christison to omit the tests of the Pharmacopœia and make this unauthorised substitution for them ? To this inquiry we can imagine that only one answer can be given, which is this : —that whereas Dr. Christison has nearly thirty times objected to the tests of the London College, that they are mere proofs of the nature of the substances and not of their purity, it would hardly be consistent in him to suffer a mere statement of colour, and absence of efflorescence to appear in his Dispensatory as proofs of chemical purity.

But this is not all: it appears from Dr. Christison's representation of the Pharmacopœia test, that a pale blue colour, occasioned by the action of excess of ammonia, is a test of purity, whereas it is well known to indicate the presence of copper.

Still further: Dr. Christison observes, that "in stating in the Edinburgh formula the ammoniacal test for indicating copper, the preliminary step for converting the oxide of iron into sesquioxide, which is quite essential, has not been mentioned." This observation is made up of candour and unfairness ; of candour, because the error is unquestionable and clearly his own ; of unfairness, because he attributes it to the College, which, as we have already shown, does not employ this test at all.

When ammonia is added in excess to a solution of protosulphate of iron, the filtered solution becomes turbid by the deposition of peroxide of iron which had been dissolved as protoxide, and attracted oxygen from the air ; hence the necessity, as mentioned by Dr. Christison, for converting the protosulphate into persulphate of iron, previously to adding the ammonia.

With respect to the composition of sulphate of iron, we may remark that Dr. Christison has committed several errors ; he says :—" The salt is composed of one equivalent of protoxide of iron, one equivelent of sulphuric acid, and six equivalents of water ($SO^3 + Fe\,O + 6\,HO$), and consequently of 36 parts of base, 40·1 of acid, and 54 of water. Hence the anhydrous salt of the Edinburgh Pharmacopœia contains about four-fifths more of real sulphate than the crystallised ; which must be attended to in prescribing it. As five of the equivalents of water are easily expelled by heat, while a much higher heat is necessary to drive off the fifth, this equivalent is considered by Professor Graham to exist in the salt as a base, so that the constitution of sulphate of iron, according to his view, is $SO^3\,FeO\,HO + 5\,Aq.$"

Now Graham's statement does not resemble the above either in form or in fact; his formula (*Elements*, p. 558) is $Fe\,O,\ SO^3,\ HO + 6\,HO$, showing that it contains not 6, but 7 equivalents of water; if, as stated, the ferri sulphas exsiccatum were anhydrous, (which it is not), then, even admitting the fact, it would be incorrect to assert that it contains any proportion whatever of itself, and yet Dr. Christison says, that "the anhydrous salt of the Edinburgh Pharmacopœia contains about four-fifths more real sulphate than the crystallized." Now, as 139 of the crystallized salt contain 76 of anhydrous sulphate of iron, 100 will give rather less than 55, instead of 80, as stated by Dr. Christison.

In the above quotation Dr. Christison has mentioned that a higher heat is required to drive off the fifth equivalent of water ; this is a mis-print for the *sixth* ; but even when thus amended the fact is not correctly stated ;

Professor Graham says (Elements, p 559), "of the 7 HO which copperas contains, it loses 6 HO at 238°, but retains 1 eq. even at 535°."

We have said that the Ferri sulphas exsiccatum of the Edinburgh Pharmacopœia is not anhydrous, as represented by Dr. Christison; it is directed to be dried at "a moderate heat;" this is not defined in the present Pharmacopœia, but in the former, *calor lenis* meant a temperature of between 90° and 110° of Fahrenheit, and we may perhaps consider the terms as synonymous, more especially as in preparing the Unguentum Citrinum the axunge is directed to be melted in the oil "with the aid of a moderate heat;" now we have, as above quoted, Professor Graham's authority for stating that sulphate of iron retains one equivalent of water, even at 535°.

Dr. Thomson has made some observations on the action of dilute sulphuric acid upon iron in relation to this salt, which we confess we do not comprehend; he observes that, "the rapid decomposition of the diluted acid by the iron must be ascribed to the sum of the affinities of the base of the acid for oxygen, and of the iron for oxygen being superior to that of the oxygen to the hydrogen of the water, which is, therefore, decomposed." Now we do not comprehend how the affinity of sulphur, which is the base of the acid, for oxygen can play any part whatever in this operation.

Like Dr. Christison, Dr. Thomson has been very unfortunate in his statements respecting the water contained in sulphate of iron; he says, "Mr. Phillips makes the proportions to be of acid 28·8, protoxide of iron 25·9, and water 45·3, or 1 eq. of acid = 40·1 + 1 of protoxide of iron = 36 + 6 eq. of water = 54; making the equiv. of the salt 130·1 $(Fe.\ \dddot{S}. + 6\ \dot{H}.)$" Now Mr. Phillips states distinctly that the salt contains 7 equivalents of water = 63, and he gives its formula FeO, $SO^3$, 7HO, instead of that above represented by Dr. Thomson, who then goes on to say, "but, according to Prof. Graham, who regards one equivalent of the water to exist as a base, it contains only 5 eq. of water;" to this we will only add that the quotation which we have given from Prof. Graham, shews that this salt contains 7 equivalents of water.

*Ferrum Tartarizatum.*—It has been shown that Dr. Christison has very unceremoniously dismissed the Edinburgh College tests of the purity of Ferri Sulphas, and substituted his own for them; it will therefore excite no surprise that he should exercise the same discretion, or the same want of it, with regard to the tests of the London College; these are thus stated with regard to *Ferri Potassio-tartras*:—"In aquâ tota liquatur. Hic liquor lacmi et curcumæ colorem non mutat. Nec a potassii ferrocyanido cæruleum fit; neque adjecto quovis alkali aut acido quicquid dejicitur. Magnes in ejus pulverem nullam vim exercet." The following is Dr. Christison's version of these statements: "Entirely soluble: without action on litmus; unaffected by ferrocyanide of potassium, acids, or alkalis: not attracted by the magnet." The reader will observe that "*in water*" is omitted after "*entirely soluble,*" *solution* before *litmus,* and *turmeric* after it; *colorem non mutat, nec cæruleum fit,* and *neque quicquid dejicitur,* are translated *unaffected.* It may indeed be admitted that the meaning of the London College has in general been preserved, but to this there is one exception; the fact being that acids *do* affect this preparation, though, as correctly stated by the London College, they do not precipitate anything from it, yet they decompose it, for if hydrochloric or sulphuric acid be added to it, ferrocyanide of potassium, will then occasion the immediate formation of Prussian blue.

Dr. Christison remarks that chemists generally assume 80 as the true number for sesquioxide of iron, there are, however, exceptions to this, for Brande, Daniell, Henry, Murray, and Thomson give 40 as the equivalent of this oxide. Dr. Christison quotes Wackenroder to prove that Ferrum tartarizatum contains both protoxide and sesquioxide of iron, and that when it is decomposed by heating with potash, black oxide of iron is separated; this is certainly an error, not only in our experience, but as proved by Soubeiran and Capitain (Journ. de Pharm., 25, 138); partial deoxidizement of the sesquioxide occurs only when the heat applied to the preparation has been too great.

Dr. Thomson has in his worst manner made woful work with the London formula for this preparation; instead of three ounces of sesquioxide of iron as directed, he says twelve ounces and a half, for eleven ounces and a half of bitartrate of potash he mentions three, and three hours digestion are stated instead of two.

*Corrosivus Sublimatus.*—The tests of the Edinburgh College for ascertaining the purity of this preparation are, " it sublimes entirely by heat; and its powder is entirely and easily soluble in sulphuric ether;" but as we are informed by the same College with respect to camphor, that " its powder evaporates entirely when gently heated," and as Dr. Christison states, that " sulphuric ether is a good solvent of camphor," allow us to enquire wherein these substances differ?

Dr. Christison, in the Edinburgh process, has directed *sulphuric acid*, instead of *commercial* sulphuric acid, to be employed, and *nitric acid* instead of *pure* nitric acid; and Dr. Thomson has ordered half a *fluidrachm* of the last mentioned acid to be used in the place of half a *fluidounce*. He also states that this salt has an *alkaline* re-action, meaning we presume an *acid* one, for it reddens litmus paper and does not alter turmeric. With respect to the crystalline form of this salt Dr. Christison states that " it is a quadrangular prism terminated by two converging planes," while Dr. Thomson describes it as a "regular tetrahedron, compressed and pointed."

*Unguentum Citrinum.*—The following is the formula for preparing this ointment in both editions of the Edinburgh Pharmacopœia:—

" UNGUENTUM CITRINUM.

Take of Pure Nitric acid, eight fluidounces and six fluidrachms;
Mercury, four ounces;
Axunge, fifteen ounces;
Olive-oil, thirty-two fluidounces.

Dissolve the mercury in the acid with the aid of a gentle heat. Melt the axunge in the oil with the aid of a moderate heat in a vessel capable of holding six times the quantity; and while the mixture is hot, add the solution of mercury, also hot, and mix them thoroughly. If the mixture do not froth up, increase the heat a little till this take place. Keep this ointment in earthen-ware vessels, or in glass-vessels secluded from the light."

It has been discovered by Dr. Christison, as he states in a note p. 530 of his Dispensatory, that, " owing to an accidental error, the proportion of olive-oil has been stated in the Pharmacopœia at 32 fluidounces, and the pure nitric acid of density 1500 has been ordered." The mistake here alluded to is afterwards explained by Dr. Christison to have arisen as follows: " An oversight has been committed in the College formula from an error made by Dr. Duncan. The proportions used by Messrs. Duncan and Flockhart are twelve ounces of nitric acid of the density 1380 to 1390, four ounces of mercury, fifteen ounces of axunge, and thirty-two ounces of olive-oil,—all taken by avoirdupois weight and none by measure;" and, adds Dr. Christison, " I have corrected the College formula accordingly." And we accordingly find the following formula substituted in the Dispensatory for that of the Pharmacopœia.

UNGUENTUM CITRINUM.

Take of Nitric acid (Dens. 1380 to 1390) nine fluidounces and a half;
Mercury, four ounces;
Axunge, fifteen ounces;
Olive-oil, thirty-eight fluidounces and a half;
Dissolve the Mercury, &c. &c.

It certainly is a remarkable fact that the errors here rectified should have appeared in two editions of the Edinburgh Pharmacopœia. Dr. Christison, after alluding to the proper temperature for mixing the ingredients, and the use of almond and rape-oil and butter, as substitutes for olive-oil, proceeds to say, " the other Colleges still follow the old faulty system; and the following will show how erroneous their proportions must be if Mr. Duncan be correct:—

| | " Nitric Acid, D. 1500. | | Mercury. | Oil and Lard. | |
|---|---|---|---|---|---|
| Edin. | 6.5 | fluidounces. | 4 ounces, Tr. | 47 | ounces by weight. |
| Lond. | 5.50 | . . . | 4 . . . | 40 | . . . |
| Dub. | 3.83 | . . . | 4 . . . | 80 | . . . |

In this attempt to exhibit the proportions of other ingredients employed by the different Colleges with a standard quantity of mercury, the first assumption is, that nine fluidounces and a half of nitric acid of specific gravity 1390, are equivalent in strength to six fluidounces and a half of sp. gr. 1500; now these six fluidounces and a half weigh 4265 grains, of which four-fifths are real acid = 3412 grains; nine fluidounces and a half of acid of sp. gr. 1390 weigh 5777 grains, containing 54·6 per cent. of real acid = 3153 grains; but if 3412 grains of real acid are equal to six fluidounces and a half of nitric acid of 1500, 3154 grains = 6 fluidounces only, instead of 6·5 as stated in the table.

The proportion of acid of the London Pharmacopœia is correctly stated, but not so the weight of the oil and lard; an imperial fluid ounce of olive-oil weighs about 402 grains, 16 fluidounces will consequently weigh 13·4 troy ounces, and these added to 24 troy ounces of lard will give only 37·4 ounces as the weight of these two ingredients, instead of 40, as given in the table. This error appears to have arisen from considering 16 fluidounces of oil as equal to 16 troy ounces, for, added to the 24 ounces of lard, they make the stated quantity of 40 ounces.

With respect to the Dublin formula, five ounces and three-quarters of nitric acid of sp. gr. 1490 are used with four ounces of mercury, four wine pints of oil, and sixteen ounces of lard. Nitric acid of 1490 contains 76·5 per cent. of real, and five ounces, six drachms = 2760 grains, therefore contain 2111 grains of real acid, which are equal to four fluidounces instead of only 3·83 as stated in the table. Taking the sp. gr. of olive-oil at 0·919, four wine pints or 64 wine fluidounces, will weigh 55·8 ounces troy, which added to 16 ounces of lard, will give 71·8 as their combined weight instead of 80 ounces.

The statement therefore exhibited by Dr. Christison, to prove " how erroneous" the other Colleges are, if his amendment of the Edinburgh and " Mr. Duncan be correct," should be altered as follows :

| | | | |
|---|---|---|---|
| Edin. | 6· fluidounces | 4 ounces T. | 47 ounces by weight. |
| Lond. | 5·50 . . . | 4 . . . | 37·4 . . . |
| Dub. | 4·00 . . . | 4 . . . | 71·8 . . . |

*Morphia.*—With respect to the composition of this alkali Dr. Christison remarks that, " the analysis most confided in, that of Liebig, represents the anhydrous alkaloid to consist of 71·36 per cent. of carbon, 17·47 oxygen, 6·56 hydrogen, and 4·61 azote; which proportions approach nearly to the following constitution in chemical equivalents—$C^{34}$ $O^{18}$ $H^6$ N = 372·08." Now it will appear by calculation, which it is not requisite to give, that the above-mentioned composition, instead of approaching that represented by the formula stated, would be more correctly denoted by $C^{36}$, $O^7$, $H^{20}$ N, while $C^{34}$ $O^{18}$ $H^6$ N represents 55·92 per cent. carbon, 38·70 of oxygen, 1·62 of hydrogen, and 3·76 of azote, instead of the composition stated by Dr. Christison; but in accordance with the incorrect formula the equivalent of morphia is stated by Dr. C. to be = 372·08.

On referring to Dr. Christison's statement of the composition of acetate of morphia, it would seem as if the figures representing the equivalents of oxygen and hydrogen had accidentally changed places in the formula which we have quoted ; for he says that acetate of morphia " is probably composed of one equivalent of base, of acid, and of water ($C^{34}$ $H^{18}$ $O^6$ N + $\bar{A}$ + Aq.) that is, 372·08 parts of morphia, 43·28 acetic acid, and 9 water."

In the formula already given, we have $O^{18}$ $H^6$, and on referring to Liebig and halving the equivalents for hydrogen and azote, he gives $C^{35}$ $H^{20}$ $O^6$ N, to which Dr. Christison's amended formula somewhat approximates; but then he has again given 372·08 as the equivalent, whereas it should be 288·08 ; it may be farther observed that, the equivalent of acetic acid is given as 43·28, instead of 51·48, which is shown at p. 8 of the Dispensatory to be its equivalent.

Under the head of Muriate of Morphia Dr. Christison has also given the more correct formula for morphia; and he states that, " the constitution of the muriate of morphia is supposed to be one equivalent of the base, one of its acid, and six of water ($C^{34}$ $H^{18}$ $O^6$ N + HCl + 6 Aq.), or

76·24, 9·66 and 14·1 per cent." Now this composition does not agree with the first, but with the second or more correct equivalent of morphia.

Dr. Thomson has also been most unfortunate in his statements respecting the composition of morphia, and has contributed his full quota towards the propagation of error; he makes the following statement: "Morphia consists of 1 eq. of anhydrous morphia = 288·23 + 2 eq. of water = 18, making the equivalent = 306·41. The anhydrous salt [alkali] consists of—

| | | | | | | |
|---|---|---|---|---|---|---|
| 34 eq. of | carbon | = | 208·08, | or | 71·36 | per cent. |
| 16 — | hydrogen | = | 16· | — | 6·56 | „ |
| 18 — | oxygen | = | 144· | — | 17·47 | |
| 1 — | nitrogen | = | 14·15 | — | 4·61 | |
| | Equiv. | = | 372·23 | | 100·00." | |

The statement that morphia consists of itself and two equivalents of water, means, we presume, that the crystals of morphia are so constituted.

The account of the number of equivalents contained in morphia, was we suppose, intended to be a copy of Dr. Christison's very incorrect representation, in which as we have already pointed out, the equivalents of oxygen and hydrogen have changed places; but in addition Dr. Thomson has slipped in ten equivalents of hydrogen, and yet finds that the equivalent is only 372·23 instead of 382·23; and, notwithstanding the addition of these ten equivalents of hydrogen, we find the same erroneous representation of the composition of 100 parts of morphia as given by Dr. Christison. It is indeed impossible not to perceive the errors of the above statements on the slightest glance; thus, the oxygen is to the nitrogen, in the above statement, as 144 to 14·15, or about 10 to 1; but in 100 parts they are given as 17·47 to 4·61 or nearly as 4 to 1.

We shall not fatigue the reader with a statement of what the composition of 100 parts of morphia is according to Dr. Thomson's incorrect representation of its atomic constitution, but merely remark that as 71·36 per cent. of carbon should be 55·9, corresponding alterations would require to be made in the quantities of the other constituents; but it would be a waste of time to correct a part when the whole is so completely erroneous.

With respect to the acetate of morphia, Dr. Thomson continues the error which he has committed, he says "acetate of morphia is a compound of 1 eq. of morphia = 372·23 + 1 acetic acid = 51·48 + 1 water = 9, making the equiv. = 432·91, or 82·95 parts of morphia + 14·15 of acetic acid and 2·55 of water = 100 parts of the salt." Adopting 372·23 as the equivalent of morphia, and 51·48 as that for acetic acid, and 9 = 1 equivalent of water, the equiv. of acetate of morphia will be 432·71, and 100 parts of it will consist of 86·02 morphia, 11·89 acid, and 2·09 water, instead of the quantities stated by Dr. Thomson.

Similar errors occur with respect to hydrochlorate of morphia. "The crystals consist of one eq. of morphia = 372·23 + 1 of hydrochloric acid = 36·42, and 6 of water = 54, making the equiv. 462·65, or 76·24 parts morphia + 9·66 acid, and 14·10 water, = 100 parts." Now, admitting as before the incorrect equivalent for morphia, 100 parts of the hydrochlorate must consist of 80·45 morphia, 7·87 acid, and 11·68 water, instead of the quantities above stated. We may observe that Dr. T.'s statement is apparently taken from Dr. Christison, and he has adopted it without appearing to know that his equivalent of morphia differs as widely from that of Dr. Christison as 372·23 from 288·08.

*Plumbi Acetas.*—In preparing this salt, the Edinburgh College have directed fourteen ounces of litharge to be dissolved in two pints of pyroligneous acid of sp. gr. 1034, and one pint of water; it is stated that 100 minims of this acid saturate 53 grains of carbonate of soda; now two pints or 40 fluidounces are equal to 19,200 minims, and consequently are capable of saturating 10,176 grains of the alkaline carbonate, equivalent to 7914 grs. of litharge, or nearly 16·5 ounces; and this is the quantity which should have been ordered instead of only 14; it therefore follows that a considerable portion of the acid must remain unsaturated with the oxide of lead.

It is stated by Dr. Christison, that when crystallized acetate of lead is heated to 320°, it loses the whole of its water, and it might be supposed that this high temperature was requisite to produce the effect; we have found however, by repeated experiments, that this salt is rendered perfectly anhydrous by exposure to the heat of steam in a porcelain vessel, and consequently even below 212°. It is further mentioned that "thirty grains of phosphate of soda and 47·66 of acetate of lead exactly decompose one another. Hence, if 48 grains or a 144th part more of the latter salt be used, the solution will be affected after filtration by a farther addition of phosphate, provided the acetate be tolerably pure." Now the difference between 48 and 47·66 is 0·34, which is almost exactly 1-140th of, instead of 1-144th of 47·66; this we are aware is minute criticism, but any statement worth making is worth correcting.

*Plumbi Nitras.*—In treating of this salt Dr. Christison observes that, "Nitrate of Lead is obtained according to the process of the Edinburgh College by dissolving lead with the aid of heat in dilute nitric acid, and crystallizing the solution by evaporation and cooling. It may be prepared equally well by dissolving litharge in the acid." It is quite evident that Dr. Christison had forgotten that the Edinburgh process, which consisted in dissolving lead in nitric acid, had been abandoned; for he could not be ignorant that the use of litharge, which he represents as equally efficient, had actually been preferred, on account of its being more so.

The following were the directions contained in the first edition of the Edinburgh Pharmacopœia for preparing

"PLUMBI NITRAS.

Take of Lead, six ounces;
Diluted Nitric Acid, six fluidounces;
Water, six fluidounces;

Mix the acid and water, and dissolve the lead with the aid of a gentle heat. Concentrate the solution, and set it aside to cool and crystallize."

It having been shown that this formula was in every respect objectionable, that to want of economy there were added improper and impracticable proportions of the several ingredients; the Edinburgh College rejected it, and the following directions are now substituted for it in the new edition:—

"Take of Litharge, four ounces and a half;
Diluted Nitric Acid, a pint;
Dissolve the litharge to saturation with the aid of a gentle heat. Filter and set the liquid aside to crystallize. Concentrate the residual liquid to obtain more crystals."

Now, if we calculate rightly, it will be impossible to saturate the acid with employing the assigned portion of litharge. The sp. gr. of the dilute nitric acid is by our experiment 1·080, a pint, or 20 fluidounces, will therefore weigh 9450 grains, and contains about 1096 grains of real acid, requiring 2273 of oxide of lead for saturation, instead of only $4\frac{1}{2}$ ounces, or 2160 grains directed for that purpose, so that about 1-20th of the acid must remain unsaturated; even with this imperfection, however, the process is a great improvement on the rejected one, and we repeat our ignorance of the cause which could have induced Dr. Christison to represent the former process as still continued, and the use of litharge merely as good as that of lead, but not as an improvement actually adopted.

*Potassæ Acetas.*—This salt is directed to be prepared as follows:—

"Take of Pyroligneous acid, a pint and a-half;
Carbonate of potash (dry), seven ounces, or a sufficiency;
Add the carbonate gradually to the acid till complete neutralization is accomplished. Evaporate the solution over the vapour-bath till it is so concentrated as to form a concrete mass when cold. Allow it to cool and crystallize in a solid cake; which must be broken up and immediately put into well-closed bottles."

The Materia Medica of the Edinburgh College contains *Potassæ Carbonas*, obtained by purifying pearlash, and among the preparations there is *Potassæ Carbonas purum;* it is stated that the former should not lose more than one-fifth of its weight, and the latter not any at a red heat. Now *dry* may mean *anhydrous*, or merely *not moist;* on the first supposition we shall examine into the sufficiency of the Potassæ Carbonas purum ordered in the first instance for the saturation of a pint and a half or 30 fluidounces of pyroligneous acid.

We have shown under *Plumbi Acetas* that 40 fluidounces of pyroligneous acid must, according to the College, saturate 10,176 grains of carbonate of soda, equivalent to 4946 grains of anhydrous carbonate of potash, 30 fluidounces will therefore require 3710 of this salt for the same purpose; the College have, however, ordered only 3360 grains, or nearly 6 drachms less than the requisite quantity.

If, however, *dry* mean merely *not moist*, the operator may then employ *Potassæ carbonas* containing 20 per cent. of water, and in this case he will require 4637 grains, or rather more than 9 ounces and 5 drachms, instead of the 7 ounces indicated. This ambiguity would have been avoided by omitting the word *dry*, or employing the term *pure*.

With respect to the solubility of this salt, Dr. Thomson states that "one fluid ounce of distilled water at 60° dissolves 504 grains; or 100 parts of it are soluble in 102 parts of water." Now, if 504 grains require a fluidounce or 437·5 grains of water for solution, 100 will be dissolved by 86·8 grains, instead of 102. Dr. Thomson proposes to detect the presence of tartrate of potash in this salt by adding to a solution one of acidulated acetate of lead, in which case, he says, a precipitate will fall soluble in acetic acid. Tartrate of lead is not, however, very readily soluble in this acid, and it would be better to employ the nitric, which immediately takes it up; indeed, the use of acidulated acetate of lead shows that the tartrate is not easily dissolved by acetic acid. Again, Dr. Thomson says that the hydrochlorates are detected by nitrate of silver, but in the employment of this test the solution must be considerably diluted, otherwise a crystalline precipitate of acetate of silver is formed, which, however, is readily distinguishable from the chloride by its solubility in a large quantity of water or a small portion of nitric acid.

*Potassæ Aqua.*—In preparing this solution the Edinburgh College have directed the use of *dry* carbonate of potash, which, as we have before remarked, may mean either the anhydrous salt, or that containing 20 per cent. of water, and this ambiguity may cause considerable variation in the strength of the preparation.

Dr. Christison has thus given the directions of the London Pharmacopœia for mixing the various ingredients employed in this solution :—" Let the lime be slaked with a little of the water in an earthen vessel; add the rest of the water; transfer the mixture immediately into a close vessel, &c. &c." It so happens that the directions for employing the carbonate of potash are entirely omitted; in the original they commence with " dissolve the carbonate of potash in half a gallon of the water," and then follow " let the lime be slaked with a little water, &c."

With respect to the Liquor Potassæ of the London Pharmacopœia, Dr. Thomson remarks, that " one pint of the solution should weigh sixteen ounces; or the specific gravity be 1·063; and it should contain 4·7 per cent. of pure potassa." A pint of water weighs 18 oz. 1 dr. 50 grains, and consequently a pint of a liquid, the density of which is 1063, must weigh 19 oz. 3 drachms, instead of only 16 oz. as stated. The statement is also improbable on mere inspection, that a liquid whose density is 1063 should contain only 4·7 per cent. of solid matter in solution. By calculation we find that the liquor potassæ must contain rather more than 5·9 per cent. of potash, and this calculation is confirmed by that of the quantity required and employed, in precipitating a given quantity of bichloride of mercury in preparing the Hydrargyri Binoxydum.

*Potassæ Carbonas.*—The Edinburgh Pharmacopœia of 1817, contained Potassæ Subcarbonas and Potassæ Carbonas, and so also does the present, but the College, " seduced by the philosophical attractions of modern chemical nomenclature," (*Preface*, *p.* xiii), have now bestowed on these preparations their correct appellations, and on this subject Dr. Christison remarks, that " great care must be taken not to confound the Carbonate of the new Edinburgh Pharmacopœia with the Carbonate of the last Latin edition, the former salt being a corrosive poison, and the latter the bicarbonate, which is not poisonous."

It appears to us that the College might, by prefixing *sub* to one carbonate, and *super* to the other, have avoided the danger described by Dr.

Christison ; and this method might have diminished the chances of poisoning, without any very material addition to the acknowledged " patchwork" of their nomenclature, which on various occasions they have needlessly increased.

The Edinburgh Pharmacopœia contains Potassæ Carbonas and Potassæ Carbonas purum ; the first of these is an article of the Materia Medica, and stated to be obtained by " lixiviating, evaporating, and granulating by fusion and refrigeration the potashes of commerce ;" this is explained to be " carbonate of potash not quite pure."

Among the preparations is the following formula for obtaining

" POTASSÆ CARBONAS PURUM.

Pure Carbonate of potash may be most readily obtained by heating crystallized Bicarbonate of potash to redness in a crucible, but more cheaply by dissolving Bitartrate of potash in thirty parts of boiling water, separating and washing the crystals which form on cooling, heating these in a loosely-covered crucible to redness so long as fumes are discharged, breaking down the mass, and roasting it in an open crucible for two hours, with occasional stirring, lixiviating the product with distilled water, filtering the solution thus obtained, evaporating the solution to dryness, granulating the salt towards the close by brisk agitation, and heating the granular salt nearly to redness. The product of either process must be kept in well-closed vessels."

The Edinburgh Pharmacopœia does not contain *crystallized* bicarbonate of potash ; the directions for preparing it being to " continue the dessication till a fine powder be obtained ;" and moreover, among the tests of bitartrate of potash, we find it stated that it is " soluble in 40 parts of boiling water," we must therefore conclude that 30, here directed for the same purpose, will not be sufficient to effect it.

Dr. Thomson, in giving the Dublin formula for preparing Potassæ Carbonas e lixivo cinere, has omitted the words " in vase argenti vel ferrî mundissimo."

*Potassæ et Sodæ Tartras.*—The London College call this salt Sodæ potassio-tartras, a name for his objections to which Dr. Christison refers to his remarks respecting antimonii potassio-tartras ; and we refer to the same article for our reply to his observations.

As to the constitution of this salt, Dr. Christison says, " it is composed of one equivalent of tartrate of potash, one equivalent of tartrate of soda, and ten equivalents of water ($2\,\bar{T} + KO + Na\,O + 8\,Aq$) ; and therefore of 132·96 parts of acid, 47·15 of potash, 31·3 of soda, and 90 of water."

It will be observed that, in his *symbolic* statement, Dr. Christison gives 8 equivs. of water to this salt, while in his *verbal* one, he assigns 10 eqs. to it ; we presume that the former, which we believe to be the correct one, was intended, for if the latter be right, then only 40·3 of nitrate of lead would be required to decompose 37 parts of this salt, whereas, we find by the Edinburgh tests, that " 37 grains in solution are not entirely precipitated by 43 grains of nitrate of lead ;" by calculation, the exact quantity of the salt of lead required appears to be 43·24 grains.

Dr. Christison has very justly criticised the mode adopted by the London College of applying the tests for detecting sulphates and chlorides, in this preparation. It ought, certainly, to have been stated that the solution of the salt should be largely diluted before adding the nitrate of silver or chloride of barium, or that the precipitates obtained by them are soluble in water and in dilute nitric acid. Under potassæ tartras the London College have directed the latter precaution to be adopted.

With respect to the composition of this salt Dr. Thomson makes the following statements: "the constituents of 100 parts of this salt, according to Schulze, are 41·3 of tartaric acid, 14·3 of potassa, 13·3 of soda, and 31·1 of water; according to Vauquelin they are, tartrate of potassa, 54 parts, tartrate of soda 46 parts; and according to Mr. Phillips, 40 parts of tartrate of potassa, 34·5 of tartrate of soda, and 25·5 of water in 100 parts. In equivalents, the crystals contain 1 eq. of tartrate of potassa = 113·63 + 1 of tartrate of soda = 97·8 + 10 of water = 90, making the equiv. 292·43." In his "table of Pharmaceutical equivalents" Dr. Thomson gives as the composition of this salt, "1 soda = 31·3, 1 potassa = 47·15, 2 tartaric acid = 132·96," making the equivalent "211·41." We may first observe, that when 113·63, 97·8, and 90 are added together, they make 301·43, and not 292·43; and, having effected this amendment, let us see, as well as we can make out, how much per cent. of its ingredients this salt must contain, according to the various statements of its composition.

| | *Schulze.* | *In equivalents.* | *Phillips.* | *Table.* | *Vauquelin.* |
|---|---|---|---|---|---|
| Tartaric acid.. | 41·3 | 44·11 | 46.5 | 62·89 | 62·87 |
| Potash ...... | 14·3 | 15·64 | 16·9 | 22·30 | 22·40 |
| Soda ........ | 13·3 | 10·37 | 11·3 | 14·91 | 14·73 |
| Water ...... | 31·1 | 29·88 | 25·3 | | |
| | 100· | 100· | 100· | 100· | 100· |

As it is generally admitted that this salt contains water, it would, we think, have been better if Dr. Thomson had stated on which of the three first analyses, if on any, he placed reliance, and had altogether rejected the two last, which not containing water, cannot represent the salt of the Pharmacopœia. It may be added, that the analysis of Schulze is evidently incorrect, for the equivalent of potash and soda being to each other as 48 to 32, and assuming the proportion of the former to be correctly stated at 14·3, that of the latter must be only 9·54, instead of 13·3.

*Potassii Sulphuretum.*—We may remark it as a very unusual circumstance, that the three Colleges agree in directing this preparation to be obtained by fusing a mixture of an ounce of sulphur and four ounces of carbonate of potash; as the Edinburgh College do not order the pure carbonate, we must conclude that they mean the less pure preparation, containing, according to their statement, 20 per cent. of water, but which we shall assume to be, like the carbonate of the London College, a sesquihydrate, containing 16 per cent. of water.

In the Materia Medica of the Edinburgh Pharmacopœia we find—

" Potassii Sulphuretum. A mixture of sulphate of potash with persulphuret of potassium. *Sulphuret of potash.*"

The error of *sulphuret of potash,* instead of the correct name of sulphuret of *potassium*, occurs in both editions of the Pharmacopœia, and is repeated by Dr. Christison in his Dispensatory ; even with this correction, the description of this compound is, we think, inaccurate. According to Berzelius, when 94 parts of sulphur are fused with 100 parts of anhydrous carbonate of potash, there are formed 31·5 of sulphate of potash, and 131 of quinto-sulphuret of potassium, the former requiring one-fourth, and the latter three-fourths of the carbonate of potash.

Dr. Christison remarks that the " College proportions amount to nearly the same number of equivalents of carbonate of potash and sulphur." If we divide 480 grains or one ounce of sulphur by 16, it will give 30 equivalents, and 4 ounces or 1920 grains of carbonate of potash divided by 70, it will give nearly 27·5 equivalents ; but this quantity of the alkaline carbonate contains 310 grains of water, which will reduce it to 23 equiv. of anhydrous carbonate, instead of 30. There is, however, a circumstance, mentioned by Berzelius, which will certainly diminish the proportion of sulphur as such ; he says that, when water is present, hydrosulphuric acid is formed and expelled with the carbonic acid gas ; he does not state what becomes of the oxygen of the decomposed water ; it is probable, however, that this also forms an acid with a portion of the sulphur, giving rise to a salt of potash ; indeed, according to Winckler, quoted by Dr. Thomson, this is actually the case.

Under these circumstances, all that it seems to be safe to state, with respect to the preparation of the Colleges, is, that it owes its power to quinto-sulphuret of potassium, which is mixed with carbonate, sulphate, and probably other salts of potash ; at any rate, it must be admitted, that the College description of its composition, not including carbonate of potash, must be incomplete, for, as already mentioned, Berzelius states that 94 require only 100 of carbonate of potash, to form quinto-sulphuret of potassium and sulphate of potash, whereas the proportions used by the Colleges are 94 of sulphur to 315 of carbonate of potash, considered as anhydrous.

*Sodæ Carbonas.*—Under this head Dr. Christison has given " a table of the respective proportions of the several acids and alkaline carbonates to be used for effervescing powders." It is as follows :—

| | *Car. Sod.* | *Bic. Sod.* | *Car. Pot.* | *Bic. Pot.* | *Sesq. Amm.* |
|---|---|---|---|---|---|
| Tartaric acid 30 grains, | 57 | 38 | 28 | 40 | 24 |
| Citric acid 30 grains, | 74 | 43 | 36 | 52 | 30 |
| Lemon-juice 1 fluidoz. | 76 | 45 | 28 | 54 | 32 |

We assume that the tartaric acid is the crystallized, containing the usual quantity of water, and in that case, 30 grains of it and 57 grains of *crystallized* carbonate of soda, are proper proportions ; we believe, however, that both these substances are usually deprived of a portion of water before employment for effervescing powders. Still, admitting the acid to be crystallized, and that Dr. Christison represents bicarbonate of soda by 84·54, the 38 in the table should be 33·6 ; again—30 grains of crystallized tartaric acid are correctly stated to saturate 28 of carbonate of potash, provided it be the pure or anhydrous preparation of the Edinburgh

Pharmacopœia, which it is not however stated to be; if, on the other hand, the less pure preparation be employed, then, as it contains 20 per cent. of water, 35 grains would be required instead of 28; the quantities of bicarbonate of potash and sesquicarbonate of ammonia are correctly assigned.

With respect to Citric Acid, we find that 30 must be anhydrous to saturate as much as 74 of crystallized carbonate of soda; but this acid cannot be obtained free from water; and in its crystalline state, 30 saturate only 61 of carbonate of soda, instead of 74. Again—if the citric acid be anhydrous, then, as stated, 30 would saturate 43 of bicarbonate of soda, but the crystals can saturate only 36 instead of 43; always supposing the acid to be anhydrous, it is correctly stated that 30 will saturate 36 of (anhydrous) carbonate of potash, 52 of bicarbonate of potash, and 30 of sesquicarbonate of ammonia.

On merely inspecting the statements of the quantity of the alkaline carbonates required to saturate a fluidounce of lemon-juice, it is evident that all but one must be wrong, and even that we believe not to be right. It will be seen that a fluidounce of lemon-juice is stated to be sufficient to saturate 28 grains of carbonate of potash, we believe 33 to be nearer the quantity; but we will admit, for the moment, that 28 is the right proportion; now this is also the quantity which saturates 30 of tartaric acid, consequently, by the doctrine of equivalents, the lemon-juice should saturate the same quantities of the other carbonates as 30 of tartaric acid; so that, supposing the quantities to be correct with regard to tartaric acid, it ought to have been stated that a fluidounce of lemon-juice saturates 57 of carbonate of soda instead of 76, 38 of bicarbonate of soda instead of 45, 40 of bicarbonate of potash instead of 54, and 24 of sesquicarbonate of ammonia instead of 32. In concluding, we recommend a very careful revision of this table.

*Sodæ Phosphas.*—Dr. Christison explains this salt to be the "triphosphate of soda and water" of Graham; we have however referred both to his paper on the phosphates (Phil. Trans. 1837) and his Elements of Chemistry, and in neither do we find any mention of such a class of salts as triphosphates; we therefore presume that Dr. Christison means tribasic phosphate of soda and water, which Prof. Graham explains in his Memoir, and also in his Elements, to be the common phosphate of soda, and the formula which Dr. Christison gives is essentially that of this salt adopted by Graham, but the former adds, "it therefore contains 62·6 parts of soda, 71·4 of phosphoric acid, and 216 of water;" the quantity of the last-mentioned constituent is, however, inaccurately stated; it should be 225 of water = 25 equivalents, instead of 216 = only 24 equivalents.

The tests employed by the Edinburgh College for ascertaining the purity of this substance are "an efflorescent salt: 45 grains dissolved in two fluidounces of boiling distilled water, and precipitated by a solution of 50 grains of carbonate of lead in a fluidounce of pyroligneous acid, will remain precipitable by a solution of acetate of lead."

We cannot imagine why a solution of the proper quantity of acetate of lead might not have been adopted instead of the troublesome method of preparing a solution of this salt from acetic acid and carbonate of lead; the acetate is much more likely to be obtained in a state of purity than the carbonate of lead; the quantity of pyroligneous acid directed for dissolving the carbonate of lead is enormous; we find that 100 minims of this acid saturate 53 grains of carbonate of soda, consequently a fluid-ounce, directed for dissolving 50 grains of carbonate of lead, is sufficient to dissolve 233 grains, or more than four times the quantity required.

It ought to have been mentioned that the precipitate occasioned by acetate of lead is entirely and readily soluble in dilute nitric acid, if unmixed with sulphate; for, not only might a portion of sulphate of lead be precipitated with the phosphate, but there are some sulphates, as ammonia alum, and soda alum, which would fulfil the conditions of precipitating the stated quantity of acetate of lead and remaining precipitable by the addition of a further quantity.

In the Dublin Pharmacopœia, this salt is directed to be prepared by decomposing 10 *parts* of burnt bone by seven *parts* of sulphuric acid, and Dr. Thomson has correctly so stated it; but, when directing the saturation with soda, he says, " then add three *pounds* and ten *ounces* of carbonate of soda (dissolved in eight parts of warm water)."

As the *parts* of sulphuric acid and bone may be pounds or ounces, or any larger or smaller weight, at the pleasure of the operator, and as any one selected would require the use of a similar one with respect to the carbonate of soda, we were somewhat surprised at finding a definite quantity of the alkaline salt to be assigned. In endeavouring to clear up the mystery, we referred to the former Dublin Pharmacopœia (1806), and there found that five *pounds* of bone, three and a half *pounds* of sulphuric acid, and three *pounds* ten *ounces* of carbonate of soda are ordered to be employed. It appears, therefore, that, by some very extraordinary mishap, Dr. Thomson has substituted three *pounds* ten *ounces* of the Pharmacopœia of 1806 for the eight *parts* of carbonate of soda of the Pharmacopœia of 1817. It is scarcely requisite to add that, by this unaccountable error, the whole directions are rendered absolutely nugatory.

Referring to the superphosphate of lime produced by the action of the sulphuric acid upon the phosphate of lime, Dr. Thomson says, its separation is ordered to be effected by " digestion in vapour, and the repeated affusions of boiling water;" we confess that we are utterly at a loss to conjecture what is meant by "digestion in vapour."

With respect to the constituents of this salt Dr. Thomson observes, that " Mitscherlich makes them 1 eq. of acid = 70·14 + 1 of soda = 31·3 + 12 of water = 108: equiv. 209·74;" where this statement is to be found we know not, but in the author's Elemens de Chimie, Vol. 3, p. 63, we find a formula, which certainly does not agree with that cited by Dr. Thomson; it is also stated, and correctly so, that " Professor Graham regards the salt as a compound of 1 eq. of acid + 2 of soda + 1 of basic water, and 24 of water of crystallization," to which it is incorrectly added, " making the equiv. 307·74 (2 Na O. + H. O. + $P^2$ $O^5$ + 24 H. O.)" for Professor Graham makes the equivalent 359·15, and in his formula we have $PO^5$, and not $P^2$ $O^5$ as above stated.

Among the tests directed to be prepared in the Edinburgh Pharmacopœia, we shall notice only two, and of these the first is Ammoniæ Oxalas; this is directed to be obtained by saturating four ounces of oxalic acid with eight ounces of sesquicarbonate of ammonia; the equivalent of oxalic acid is 63, and that of the ammoniacal salt, 59, considering it as containing one equivalent of alkali; consequently four ounces, or 1920 grains, of the acid require for saturation only 1798 of the 3840 grains, or eight ounces, directed to be used, without any occasional trial of the state of saturation.

On referring to Dr. Christison's Dispensatory, (p. xx.) we think we discover the cause of this mistake, he says " the process for oxalate of ammonia is a case of simple decomposition. Carbonate of ammonia and oxalic acid being brought together in their equivalent proportions." In p. 112, Dr. C. represents the following as the formula for the ammoniacal salt, " $2\,H^3\,N + 3\,CO^2 + 2\,HO = 1$ sesquicarbonate of ammonia," and on this view of the subject, and adopting Dr. Christison's equivalents, the number of this salt is 118·66, and consequently in directing 3840 grains, the excess would appear to amount to only about 224 grains. It is therefore evident that the process for preparing oxalate of ammonia was never submitted to experiment, and the erroneous directions for its preparation arose from supposing that the double equivalent of sesquicarbonate of ammonia contained only one instead of two equivalents of the alkali.

We do not speak from theory, it was evident that the experiment would occupy but a few minutes and we made it,—and we found that, when equal weights of oxalic acid and sesquicarbonate of ammonia were mixed with distilled water, the oxalic acid was so perfectly saturated as not to affect litmus paper, but on the contrary, that the solution turned turmeric paper brown, and yielded ammonia when heated, as was perceptible both to the smell and to turmeric paper.

*Barytæ Nitras.*—The instructions are that, " this salt is to be prepared like the muriate of baryta, substituting pure nitric acid for the muriatic acid." We are therefore to *substitute* half a pint of pure nitric acid of sp. gr. 1·5 for the same quantity of pure muriatic acid of sp. gr. 1·17; thus using the same measure of these very different acids, with the same quantity of carbonate of barytes; half a pint of the muriatic acid is capable of decomposing about 9½ ounces of the carbonate of barytes, whereas the same measure of the nitric will require 20 ounces for saturation; it is therefore evident that the College should have assigned the proper proportions acid and carbonate, or at any rate have directed the nitric acid to be saturated.

In concluding our examination, we may state that the Edinburgh Pharmacopœia contains numerous points of minor importance, requiring attention and amendment, in addition to the graver errors which we have detected and exhibited. With respect to the Dispensatories, it must be evident that these works imperiously demand the most careful revision and correction to render them either safe guides to the community, or worthy of the reputation of their authors.

