# Errata

**Published:** 1845-01

**Authors:** 


					, u..v?..tUtiii.ivo, cue impci lections."'
Page 500, line 15 from top, for " and we repeat our ignorance of the cause which
could have induced"?read, " though, as already pointed out, forgetfulness has
occasioned."
1
J

				

